# A Temporal Learning Approach to Inpainting Endoscopic Specularities and Its Effect on Image Correspondence

**DOI:** 10.1016/j.media.2023.102994

**Published:** 2023-12

**Authors:** Rema Daher, Francisco Vasconcelos, Danail Stoyanov

**Affiliations:** Wellcome/EPSRC Centre for Interventional and Surgical Sciences (WEISS), University College London, Gower Street, London, WC1E 6BT, UK

**Keywords:** 68T07, 65D19, 68T45, 41A10, Surgical AI, Surgical Data Science, Endoscopy, Specular highlights, Inpainting, Temporal GANs, Transformers

## Abstract

Video streams are utilised to guide minimally-invasive surgery and diagnosis in a wide range of procedures, and many computer-assisted techniques have been developed to automatically analyse them. These approaches can provide additional information to the surgeon such as lesion detection, instrument navigation, or anatomy 3D shape modelling. However, the necessary image features to recognise these patterns are not always reliably detected due to the presence of irregular light patterns such as specular highlight reflections. In this paper, we aim at removing specular highlights from endoscopic videos using machine learning. We propose using a temporal generative adversarial network (GAN) to inpaint the hidden anatomy under specularities, inferring its appearance spatially and from neighbouring frames, where they are not present in the same location. This is achieved using in-vivo data from gastric endoscopy (Hyper Kvasir) in a fully unsupervised manner that relies on the automatic detection of specular highlights. System evaluations show significant improvements to other methods through direct comparison and ablation studies that depict the importance of the network’s temporal and transfer learning components. The generalisability of our system to different surgical setups and procedures was also evaluated qualitatively on in-vivo data of gastric endoscopy and ex-vivo porcine data (SERV-CT, SCARED). We also assess the effect of our method in comparison to other methods on computer vision tasks that underpin 3D reconstruction and camera motion estimation, namely stereo disparity, optical flow, and sparse point feature matching. These are evaluated quantitatively and qualitatively and results show a positive effect of our specular inpainting method on these tasks in a novel comprehensive analysis. Our code and dataset are made available at https://github.com/endomapper/Endo-STTN.

## Highlights

• A temporal learning-based solution to endoscopic specular highlight removal.
• A pseudo ground truth dataset for unsupervised training and quantitative evaluation.
• A quantitative and qualitative evaluation of our approach.
• An evaluation of the effect of inpainting specular highlights on downstream tasks.

## Graphical abstract

**Figure:**
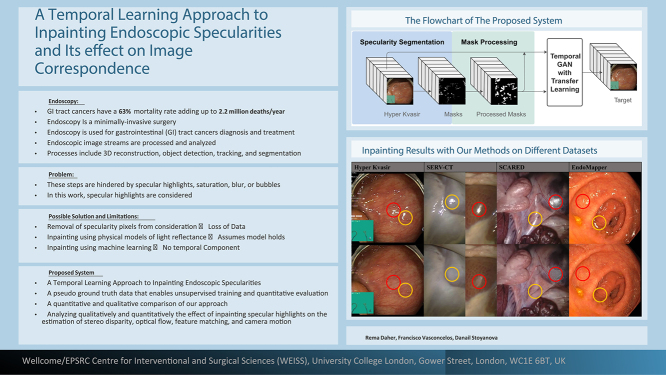

## 1 Introduction

Specular highlights in digital images commonly occur with discrete light sources. They present a serious problem in applications that rely on image processing and analysis, such as depth perception, localisation, and 3D reconstruction (Tao et al., 2015, Ozyoruk et al., 2021). These highlights not only occlude important colours, textures, and features but also act as additional features that may be falsely interpreted as being characteristic of the scene.

**Fig. 1:**
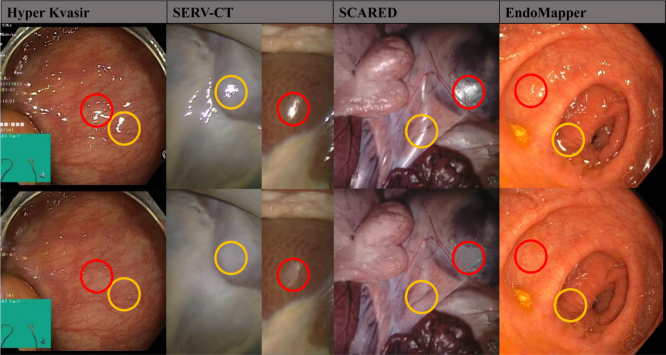
Specular highlight inpainting results with our proposed method. Our model is trained on a portion of the Hyper Kvasir dataset (upper and lower gastric endoscopy). Provided results are for unseen Hyper Kvasir images and other datasets without any additional fine-tuning, including in-vivo colonoscopy (EndoMapper) and ex-vivo porcine laparoscopy (SERV-CT, SCARED). The yellow circles highlight important inpainted areas where the spatial and temporal components were utilised to recover occluded textures. Red circles show areas where some details were lost with inpainting. For more inpainting examples on both in-vivo endoscopy and ex-vivo porcine laparoscopy refer to supplementary material and Fig. 5, Fig. 6, Fig. 7.

The effect of specular highlights can be especially harmful when dealing with surgical imaging. Some procedures that are affected by specularities include dermatological imaging (Madooei and Drew, 2015), cervical cancer screening (Kudva et al., 2017), laparoscopy (Stoyanov and Yang, 2005), endoscopy (Ali et al., 2021), and cardiac imaging (Alsaleh et al., 2015).

This paper focuses on the removal of specular highlights and its effect on other image processing algorithms in the context of minimally invasive surgery (MIS) and diagnostic procedures (MISD).

To be able to diagnose, treat, or take biopsies during MISD, a clear understanding of the surgical scene and the ability to analyse it is of great importance, whether the analysis is done solely visually by the surgeon or with the assistance of computer vision (Chadebecq et al., 2023). However, specular highlights highly obscure the visibility of the scene. Specifically, the occlusions caused by specular highlights have a detrimental effect on the surgeons’ ability to detect anomalies and treat them. According to the study done by Vogt et al. (2002), surgeons selected images with inpainted specular highlights as higher or better quality.

These highlights also negatively affect the success of numerous MISD computer vision tasks. These tasks include providing a better depth perception, object recognition, motion tracking, 3D reconstruction, localisation, etc. For instance, Oh et al. (2007) show how specular highlights interfere negatively in uninformative frame classification and thus remove specular pixels from consideration. In addition, Hegenbart et al. (2011) show that specularities have a negative impact on celiac disease classification. As for polyp analysis, specular highlights degrade the performance of polyp detection, segmentation, and histology estimation (Prasath, 2016, Zhang et al., 2018, Urban et al., 2018, Sánchez et al., 2017).

Unlike physical object occlusions (e.g. caused by surgical instruments), specular highlights have irregular motions and shape patterns, since they change position, disappear, and appear according to tissue properties, light incidence, and camera position. Therefore, several approaches have been developed to specifically target the detection, modelling, and removal of specular highlights. One approach is to segment image regions where specularities are present and remove them from further analysis (Oh et al., 2007), however, this may discard useful information from the images. Another approach is to mathematically model the physical properties of these highlights (Hao et al., 2020), which can help in estimating the 3D shape and deformation of visualised anatomy. This, however, is a very complex task that involves accurately modelling light reflection and scattering from the visualised tissue and properties of the camera light source. Others have worked on improving computer vision tasks such as depth estimation to be less susceptible to specularities (Mathew et al., 2020). Finally, the solution that we will focus on is to inpaint these highlights and fill in the missing information.

The positive effect of inpainting on various computer vision tasks in endoscopy is utilised in the literature. For instance, when Stoyanov and Yang (2005) inpainted specularities, 3D reconstruction generated more stable results. Ozyoruk et al. (2021) also use inpainting before estimating depth for their 3D reconstruction pipeline. Heart surface tracking is also improved with inpainted specularities (Gröger et al., 2005). Moreover, a substantial improvement is detected with the realignment of colour channels when using inpainted specular highlights (Arnold et al., 2010). Furthermore, specular highlight inpainting also improves tool segmentation (Saint-Pierre et al., 2011) and polyp localisation (Bernal et al., 2013, Bernal et al., 2015).

There are two classic approaches to inpainting specular highlights: spatial–temporal patch searching, and diffusion methods. The first one attempts at finding image patches containing the occluded regions in neighbouring video frames where the specularities are not present in the same location (Zeng et al., 2019, Guo et al., 2016, Newson et al., 2014, Stehle, 2006). Whereas diffusion methods propagate information from the neighbourhood of specular regions within the frame using interpolation (Arnold et al., 2010). However, both these classic approaches heavily rely on manually tuned parameters, give low-quality results, and fall for local minima. Therefore, learning-based approaches have recently been proposed as a more reliable solution (Ali et al., 2021, Siavelis et al., 2020, Funke et al., 2018).

In this paper, a machine learning approach is used to inpaint specular highlights in gastrointestinal tract (GI) endoscopy videos by training a temporal generative network. Endoscopy is a very important procedure under the umbrella of MIS and MISD. Endoscopy has been used to diagnose GI tract cancers, perform surgery to treat such problems or carry out biopsies. The human GI tract cancer has a 63% mortality rate, which accounts for 2.2 million deaths per year (Borgli et al., 2020). Being able to diagnose such cancers early on would save many by reducing these numbers significantly. These characteristics make endoscopy an important routine procedure.

Endoscopic scenes are challenging in computer vision due to their few distinctive texture and geometry features. Another challenge is the uniqueness of endoscopic specular highlights in terms of shape, movement along frames, and conditions for appearance. This makes it hard to learn and model such artefacts, which leads to a lack of ground truth data. Since data is a key component of any learning-based approach, synthetic data has been developed but is still not very realistic (Ozyoruk et al., 2021). In addition to that, with the limited real data in general in the medical field, a clear data shortage arises. All these challenges make this problem different from the usual video inpainting problem.

The available learning-based endoscopic specularity inpainting methods have so far only been focused on single-frame inpainting. In this paper, a learning-based approach with a temporal component will be adapted from general video inpainting and used for the task at hand. This method can take into consideration the relation between consecutive frames as well as neighbouring pixel relations. Specifically, an attention-based temporal generative adversarial network (GAN) is used to inpaint specular highlights, which in turn enhances endoscopic video streams.

In addition, to deal with the limited training data and the absence of ground truth to the occluded textures behind specular highlights, a “pseudo” ground truth was generated. The pseudo ground truth is achieved by translating the specular mask within the same frame and then removing any overlaps with the original mask as can be seen in Fig. 3. These generated pseudo masks dictate the regions the model will inpaint. This way, the translated and processed masks now cover regions that have known textures. The paired data that is obtained at this point is called “the pseudo ground truth dataset” since the known textures used are not the original ones that the specular highlights occlude.

Even though our method is trained on GI endoscopy data, experimental evaluation is also extended to laparoscopic ex-vivo porcine data. This is performed to assess the model’s effect on datasets with available 3D reconstruction ground truth. The tested datasets include in-vivo GI endoscopic data from the publicly available Hyper Kvasir dataset (Borgli et al., 2020), colonoscopic data from the EndoMapper dataset (Azagra et al., 2022), and laparoscopic ex-vivo porcine data (SERV-CT Edwards et al., 2022, SCARED Allan et al., 2021). Some results on the tested datasets are shown in Fig. 1. It is worth mentioning that the system was trained only on Hyper Kvasir data and tested on all 4 datasets.

To the best of our knowledge, this is the first work in endoscopic highlight removal that employs a deep learning-based solution with a temporal component, which effectively exploits occluded texture information from different frames within the video. Another contribution lies in an in-depth, comprehensive analysis of the effect of the specular highlight processing pipeline on a diverse range of tasks that are typically used in vision-based localisation, mapping, and reconstruction. The considered tasks in this paper include sparse feature matching, optical flow, camera motion estimation, and stereo disparity calculation. While previous works have studied some of these tasks in isolation (Ali et al., 2021, Kaçmaz et al., 2020, Stoyanov et al., 2005), we assess the specular highlight processing pipeline for all tasks. Furthermore, the existing evaluation of feature matching and optical flow relies on synthetic image warping that ignores the characteristic effects of specular highlights in continuous videos such as shape change and disappearance/appearance. To overcome this we use datasets with available geometry ground truth (SCARED, SERV-CT). We also use this analysis for further comparisons with other methods.

The contributions of the proposed system can be summarised as follows:

• A novel solution to specular highlight removal in endoscopic videos. This is achieved with a temporal learning-based method as opposed to previous approaches that either ignore the temporal component or rely on parameter-heavy patch-based approaches. Our model was adapted from the general-purpose object inpainting approach of Zeng et al. (2020).
• The generation of pseudo ground truth data for the Hyper Kvasir dataset that enables effective unsupervised training of our model, as well as quantitative evaluation in unseen images without relying on synthetic image warping.
• A quantitative and qualitative comparison of our approach against both learning-based and classic inpainting approaches, demonstrating the positive effect of temporal learning.
• Analysing qualitatively and quantitatively the effect of inpainting specular highlights, using various methods, on the estimation of stereo disparity, optical flow, feature matching, and camera motion.

The rest of the paper is divided into Section 2, which includes a discussion of the related work, Section 3, which details the proposed system, followed by Section 4, which describes the experiments performed to achieve the results, that are presented and analysed in Section 5.

## 2 Related work

This section will depict the work related to handling specular highlights in endoscopy, general-purpose video inpainting methods, and also the effect of specular highlight removal on computer vision tasks in endoscopy.

### 2.1 Endoscopic specular highlight processing

Four different approaches have been used to handle specular highlights in endoscopy. The first one and the most simple is to perform highlight detection or segmentation and exclude either image regions or entire frames from further processing. To remove entire frames, various quality metrics could be used, such as those presented in Oh et al., 2007, Menor et al., 2016 and Chikkerur et al. (2011). However, such an approach results in data loss where occluded texture information is discarded.

Another approach is to represent specular highlights using a mathematical parametric model. After modelling these highlights, depth, as well as motion estimation, can be performed from the specular highlight characteristics (Hao et al., 2020). However, this does not eliminate the problem of data loss and occluded information which is very important in applications such as polyp detection and feature matching. Additionally, these can be complex models that require priors on the properties of the camera, light source, and visualised tissue, which can be difficult to obtain in a dynamic environment.

The third approach is making computer vision tasks in endoscopy more robust and less susceptible to specular highlights. For learning-based tasks, this is done by modifying their training, architecture, or loss functions. For instance, Mathew et al. (2020) use augmentation in training, which improves depth estimation. Another example is the system proposed by Barbed et al. (2022), where a loss function is added to encourage the model to ignore specularity regions in feature extraction.

The fourth way of handling specular highlights is to inpaint the affected regions with the underlying texture. Some methods rely on an optimisation formulation that searches for well-matching patches or pixels to the occluded ones in the rest of the image or previous frames and uses it as a replacement (Guo et al., 2016, Shen et al., 2020, Chao et al., 2021, Qian et al., 2022, Nie et al., 2023). In these methods, motion fields are assumed to be homogeneous and thus do not perform well with complex motions. These methods are also considered computationally expensive. An alternative technique is based on diffusion, which smoothly propagates the information in the neighbouring image regions to highlights through interpolation (Arnold et al., 2010, Zhang et al., 2022, Bidokh and Hassanpour, 2023). Both these approaches are limited to local structures and do not capture the global environment. They also produce low-quality results as the missing regions increase in size and have manually tuned parameters. Automated tuning of these parameters has been proposed in Yousaf and Qin (2014) using a two-layer feed-forward neural network. However, the ground truth data is manually chosen based on visual analysis, which adds some inaccuracies. A third inpainting technique relies on data-driven machine learning to automatically generate inpainted specularities such as in Ali et al., 2021, Siavelis et al., 2020, Funke et al., 2018, García-Vega et al., 2022a, Monkam et al., 2021 and García-Vega et al. (2023).

Most learning-based approaches to inpainting rely on generative model architectures. Endoscopic videos of the colon, liver, and stomach have been inpainted with an end-to-end cycle GAN with a self-regularisation loss (Funke et al., 2018). Siavelis et al. (2020) use GANs to inpaint laparoscopic cholecystectomy images. Cycle GANs have been proposed to restore gastro-oesophagal endoscopic specular highlights with an L1 and edge-aware losses (Ali et al., 2021). The model is initialised with the weights of the pre-trained model on the Places 2 dataset (Zhou et al., 2017). It is then retrained using a bottleneck approach and tested on endoscopic videos of the oesophagus and pyloric region of the stomach. Finally, Rodríguez-Sánchez et al. (2017) use a Convolutional Encoder-Decoder network to segment and then inpaint GI tract endoscopic specular highlights by relying on limited spatial information segmented by the encoder. However, all these approaches rely on spatial information and do not take advantage of the temporal relation between the video frames.

Reliable ground truth for highlight removal in real surgical videos does not exist for any of the analysed procedures, and therefore the evaluation of the above methods has some limitations. Some works only provide visual results for qualitative interpretation (Siavelis et al., 2020) and others perform quantitative evaluations on non-surgical data (Rodríguez-Sánchez et al., 2017). To perform a quantitative evaluation on the targeted surgical domain, one could rely on self-training approaches (Funke et al., 2018) to generate artificial paired data, but this may produce unrealistic images. Alternatively, Ali et al. (2021) add randomly placed masks to non-occluded image regions so that inpainting can be compared against a known tissue pattern. However, random masks do not capture realistic temporal information in continuous videos. In this paper, we generate a non-random pseudo ground truth for endoscopic specular highlight masks that is appropriate for continuous video analysis, both during training and evaluation of our model.

### 2.2 Temporal video inpainting

Temporal video inpainting has been applied to applications such as re-targeting videos (Kim et al., 2019), restoring saturated areas (Lee et al., 2019), and removing objects from videos (Tukra et al., 2021). In fact, all the other state-of-the-art temporal video inpainting methods that will be discussed in this section focus on object or synthetic random shape removal from diverse videos including people, faces, animals, vehicles, and other common objects. The aim of these methods is to create an inpainting technique that works on any application. However, in our application domain, more challenges need to be addressed and taken into consideration. These include the discontinuous movement of specular highlights throughout the videos due to the composition of the surfaces of the GI environment and the angle of the incident light. Another challenge is related to the few distinctive features, colours, and textures, as well as the absence of geometric structures in endoscopic videos. Most importantly, video inpainting methods have benchmark annotated ground truth datasets that can be used for training and testing, whereas, this is far from the case in the endoscopic field, where data is limited and the ground truth is hard to obtain.

The proposed architectures for temporal inpainting that mostly focus on object or synthetic random shape removal from diverse videos include 3D and 2D CNNs with a temporal component (Wang et al., 2019), which are improved on in Xu et al. (2019) and Zhang et al. (2019) by estimating appearance simultaneously with optical flow. For coherency in the temporal domain, Kim et al. (2019) use Recurrent Neural Networks and Chang et al. (2019) inpaint free-form videos using temporal shift modules (Lin et al., 2019) and temporal SN-PatchGAN (Yu et al., 2019). However, all these methods do not use information from distant frames (Zeng et al., 2020). And even with the extension of Gao et al. (2020) on the method in Xu et al. (2019), where they use edge information for flow estimation and add three distant frames, there are still very limited distant frames taken into consideration.

To address this issue, attention models have been used. Lee et al. (2019) and Oh et al. (2019) assume homogeneous motions and perform frame-wise processing of videos without any temporal coherency optimisation; only post-processing is used, which is time-consuming and unreliable with a high number of artefacts. Zeng et al. (2020) address these problems by learning a joint Spatial-Temporal Transformer Network (STTN) using a multiscale attention module with a similar logic as the traditional method that searches for patches spatially and temporally to fill in the specified occlusions.

There are a few recent methods that build on STTN to generate higher resolution inpainted textures. These include introducing an Aggregated Contextual-Transformation GAN (AOT-GAN) (Zeng et al., 2022), combining 3D CNNs with a temporal shift and align module (Zou et al., 2021), and introducing a Deformable Alignment and Pyramid Context Completion Network with temporal attention (Wu et al., 2021). Additionally, more complex occlusions can be handled with a Decoupled Spatial-Temporal Transformer with a hierarchical encoder (Liu et al., 2021).

### 2.3 Specular highlight removal effect on computer vision tasks in endoscopy

Kaçmaz et al. (2020) analyse the effect of specular highlight inpainting on the classification and detection of polyps in colonoscopy. Other works evaluate tasks related to localisation and reconstruction and are more closely aligned with the work in this paper. For example, Stoyanov et al. (2005) perform a qualitative analysis of the effect of specular highlights on depth estimation and 3D reconstruction. In addition, Ali et al. (2021) evaluate the effect of inpainting on optical flow and feature matching, providing a quantitative analysis of the results on synthetically generated data. Known geometric and photometric transformations as well as scaling and Gaussian blur were applied to frames to generate a pseudo ground truth. However, this ignores the innate characteristics of specular highlight patterns, such as discontinuity, sudden appearance and disappearance, and change in size and shape. Finally, the above-proposed methods do not use any temporal component.

In this paper, we analyse the effect of specularity inpainting both qualitatively and quantitatively on stereo disparity, optical flow, feature matching, and camera motion estimation. The disparity evaluation uses the ex-vivo porcine data with depth ground truth (SERV-CT dataset Edwards et al., 2022). Effects on optical flow and feature matching are evaluated by using them for relative camera motion estimation, which can be compared against robot kinematics ground truth on the SCARED dataset (Allan et al., 2021).

## 3 Proposed method

At the time of creating the proposed system, the system presented by Zeng et al. (2020) was the state-of-the-art method in temporal video inpainting. In parallel to STTN, Tukra et al. (2021) developed another generative network to fuse spatial and temporal information.

In this paper, the openly available system presented by Zeng et al. (2020) is adapted to fit the endoscopic specularity application. The adaptation was performed by training STTN on specularity masks with pseudo ground truth instead of object occlusions with temporal random masks that can be very different in terms of temporal coherency and continuity. These temporal random masks are generated by creating a random shape for every video sequence and moving its location along the frames using cubic Bezier curves to ensure a smooth movement along frames (Zeng et al., 2020). The pseudo ground truth dataset had to be generated and processed to create an end-to-end solution. Finally, transfer learning was utilised to generate better results.

Since the proposed model relies heavily on STTN (Zeng et al., 2020), a summary of its architecture is provided, followed by an explanation and description of the modifications made.

### 3.1 STTN summary

First, masked video frames $X_{1}^{T}≔\left\{X_{1},X_{2},\ldots ,X_{T}\right\}$, their corresponding masks $M_{1}^{T}≔\left\{M_{1},M_{2},\ldots ,M_{T}\right\}$, along with the target frames $Y_{1}^{T}≔\left\{Y_{1},Y_{2},\ldots ,Y_{T}\right\}$ are used to train the network, where T is the frame length and (H, W) is the frame size. This method follows a similar intuition to traditional methods that search for spatial–temporal patches that require inpainting. This model learns to inpaint missing regions by searching spatially and temporally through neighbouring $X_{t-n}^{t+n}$ and distant uniformly sampled frames $X_{1,s}^{T}$ at a rate of s. The problem can be formulated as a “Multi-to-Multi” problem, and using the Markov assumption, (Hausman and Woodward, 1999), the real data distribution $p\left(Y_{1}^{T}|X_{1}^{T}\right)$ can be estimated from the output ${\overset{ˆ}{Y}}_{1}^{T}$ of the learnt mapping function:

(1) $$p\left({\overset{ˆ}{Y}}_{1}^{T}|X_{1}^{T}\right)=\overset{T}{\underset{t=1}{\prod }}p\left({\overset{ˆ}{Y}}_{t-n}^{t+n}|X_{t-n}^{t+n},X_{1,s}^{T}\right)$$

A learning-based model is needed here to ensure temporal consistency and coherency between all frames. STTN includes a spatial–temporal transformer with multiple layers and multiple heads, sandwiched by a frame-level encoder from the left side and a decoder from the right. The encoder and decoder are made up of 2D convolution layers and are used to encode features from frames and back again.

**Fig. 2:**
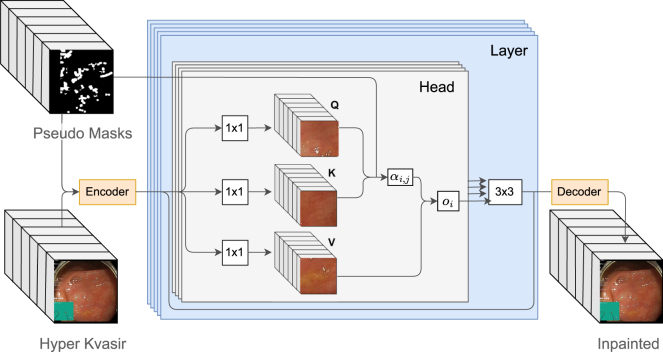
The flowchart of the network architecture of the proposed system with our pseudo ground truth training. The architecture is a multi-head multi-layer transformer with embedding, matching and attending steps.

The spatial–temporal transformer as shown in Fig. 2 has multiple heads responsible for running the transformer across different scales to handle complex motions. For example, large patches that are found in previous frames or in the same frame can be used to inpaint backgrounds and smaller ones can be used to find detailed correspondences in the foregrounds. As for the multiple layers, they help in improving the attention output by taking advantage of updated region features. Empirical studies showed that a number of 8 layers is optimal.

The transformer is made up of three steps, which are embedding, matching and attending. In the embedding stage, the features outputted by the frame level encoder are mapped into query $q_{i}$ and key–value pair $\left(k_{i},v_{i}\right)$ embedding using 1 × 1 2D convolutions $M_{q}\left(f_{i}\right),\left(M_{k}\left(f_{i}\right),M_{v}\left(f_{i}\right)\right)$, where $f_{i}$ are the encoded features and 1≤i≤T.

In the matching stage, which is carried out in every head, spatial patches that will be used to inpaint the specular region are extracted from each frame’s $q_{i}$ and $\left(k_{i},v_{i}\right)$, as $p_{i}^{q}$ and $\left(p_{i}^{k},p_{i}^{v}\right)$, respectively. With the patch size being r1×r2×c, the similarity between the ith and jth patches becomes (1≤i,j≤N):

(2) $$s_{i,j}=\frac{p_{i}^{q}\cdot {\left(p_{j}^{k}\right)}^{T}}{\sqrt{r1\times r2\times c}}$$

The attention weights are then produced for every found patch using the normalised $s_{i,j}$ and a softmax function, where N=T×h/r1×w/r2:

(3) $$\alpha_{i,j}=\left\{\begin{array}{cc} \exp\left(s_{i,j}\right)/\overset{N}{\underset{n=1}{\sum }}\exp\left(s_{i,j}\right),\; & p_{j}\in \Omega \\ 0,\; & p_{j}\in \overset{¯}{\Omega } \end{array}\right)$$

where Ω and $\overset{¯}{\Omega }$ are the unoccluded and missing regions, respectively.

In the attending stage, we can get the output from the query using the attention weights:

(4) $$o_{i}=\overset{N}{\underset{j=1}{\sum }}\alpha_{i,j}p_{j}^{v}$$

Subsequently, the output from all found patches is pieced together. The results from the various heads get concatenated and inputted into a residual block to maintain frame-wise context.

The optimisation objective function used is:

(5) $$L=\lambda_{hole}\cdot L_{hole}+\lambda_{valid}\cdot L_{valid}+\lambda_{adv}\cdot L_{adv}$$

such that L1 losses between generated and original frames are used for holes and valid regions with ⊙ being the element-wise multiplication, which gives the following functions:

(6) $$L_{hole}=\frac{{\left\|M_{1}^{T}⊙\left(Y_{1}^{T}-{\overset{ˆ}{Y}}_{1}^{T}\right)\right\|}_{1}}{{\left\|M_{1}^{T}\right\|}_{1}}$$

(7) $$L_{valid}=\frac{{\left\|\left(1-M_{1}^{T}\right)⊙\left(Y_{1}^{T}-{\overset{ˆ}{Y}}_{1}^{T}\right)\right\|}_{1}}{{\left\|\left(1-M_{1}^{T}\right)\right\|}_{1}}$$

With D as the discriminator, the adversarial loss is also represented as follows:

(8) $$L_{adv}=-E_{z∼P_{{\overset{ˆ}{Y}}_{1}^{T}}\left(z\right)}\left[D\left(z\right)\right]$$

Empirically, constant values in these functions have been suggested in Zeng et al. (2020): $\lambda_{hole}=1,\lambda_{valid}=1$, and $\lambda_{adv}=0.01$.

Adopted from T-PatchGAN (Chang et al., 2019), the discriminator loss is as follows:

(9) $$L_{D}=E_{x∼P_{Y_{1}^{T}}\left(x\right)}\left[ReLU\left(1-D\left(x\right)\right)\right]+E_{z∼P_{Y_{1}^{T}}\left(z\right)}\left[ReLU\left(1+D\left(z\right)\right)\right]$$

### 3.2 Modifications

To adapt STTN to specularity removal in endoscopic videos, some modifications had to be made. First, their model is trained on inpainting temporal random masks in diverse videos, thus the input to the training is the diverse videos and randomly generated masks with frame-ensured continuity. However, for specularity removal in endoscopy, endoscopic videos are used along with specularity masks that were pre-processed to create a pseudo ground truth for the occluded regions. A pseudo ground truth generated in an unsupervised manner is needed given that manual ground truth in high volumes is not available and very challenging to obtain.

**Fig. 3:**
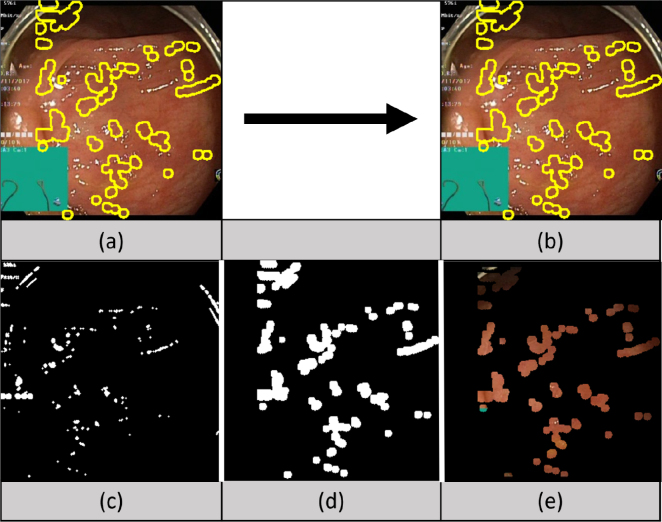
The original video frame (a) is inpainted (b) using a pseudo mask. To create this pseudo mask the original video frame is first segmented generating a specularity mask (c), which is then processed (d). From this pseudo mask we can also define a pseudo ground truth (e).

To generate a pseudo ground truth dataset, the input video streams should be accompanied by specularity masks as well as a pseudo ground truth texture behind these masks. Frames are first extracted from endoscopic videos (24 frames per second). After that, the specularity masks are generated from the frames using the segmentation method proposed by El Meslouhi et al. (2011). This segmentation technique is based on the Dichromatic Reflection Model (DRM) and makes use of the chromatic characteristics of the specular highlights. Using an unofficial implementation of this technique (https://github.com/jiemojiemo/some_specular_detection_and_inpainting_methods_for_endoscope_image), the masks are dilated after segmentation using a diamond morphological structure (Fig. 3-(c)). Other segmentation methods could potentially be applied, but alternatives either have similar performance or are not yet open source such as learning-based methods.

After segmentation, the masks were additionally dilated using an elliptical morphological structure to cover the dark rings that usually occur around specularities as shown in Fig. 3-(d). An elliptical morphological operator was used instead of a rectangular or a cross-based one since, experimentally, we found that the elliptical operator was the most realistic, whereas others were found to change the shape of specularities by making them resemble structures with perpendicular lines such as rectangles.

The masks are then processed by translating them within the same frame in a way that would make them cover up visible/unoccluded texture that would act as a pseudo ground truth. The resulting masks are denoted as $Masks_{Trans}$. To do that, the specularities were translated to the right within the same frame and any overlap between their current and old specularity locations was not taken into consideration.

After the masks are translated and processed, they move to a position that was originally without specularity and thus the texture behind the generated masked regions would be known. That way paired data is achieved, but since it is not the real occluded textures of the original specularities, we call these textures “pseudo ground truth” making the name of the dataset “the pseudo ground truth dataset”. As can be seen in Fig. 3, to generate the pseudo ground truth, whole frame specular masks are used, with a fixed translation for an entire sequence. Thus, mask changes over time are realistic, but they are offset within the same frame from the real mucosa shape that generated them.

Note that Fig. 3 uses the pseudo mask for evaluation and thus slightly differs from the training mask. The difference comes from switching the order of translation and dilation. The mask in Fig. 3-(c) is translated first, and then the overlap with the original image dilated using a ball structuring element is removed. After that the mask is dilated to generate Fig. 3-(d).

**Fig. 4:**
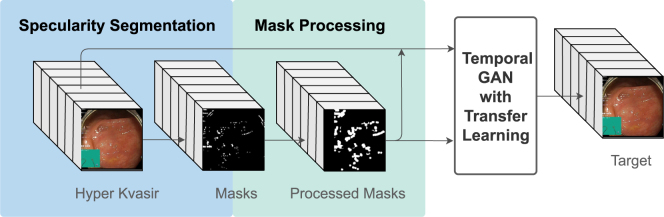
The flowchart of the proposed system is comprised of a specularity segmentation step, where specularity masks are generated from the input video dataset. After that, the masks are processed (moved to new unoccupied locations) to create a pseudo ground truth dataset. The pseudo ground truth along with the original video of the Hyper Kvasir dataset are used to train a temporal GAN, which is initialised by a model trained on temporal random masks.

In summary, input training masks were modified from temporal random masks of the STTN baseline method to segmented and processed specularity masks, $Masks_{Trans}$. Moreover, training on temporal random masks with endoscopic videos was used as an initialisation phase followed by fine-tuning on $Masks_{Trans}$. This transfer learning technique was performed to help the model obtain a more accurate initial guess. Input frames were also cropped and resized to 288 × 288 as opposed to 432 × 240 (used by STTN) since having a uniform square size for the different video frames in our pseudo ground truth dataset made it more efficient to deal with the data. Thus, a change also had to be made to the sizes list of the patches to be searched for, which in turn affected feature sizes. The proposed system then becomes as shown in Fig. 2, Fig. 4.

## 4 Experiments

For training and testing, an NVIDIA V100-DGXS was used. The endoscopic videos used for data generation are the videos of the Hyper Kvasir dataset (Borgli et al., 2020). The Hyper Kvasir videos are raw videos of routine clinical examinations of the GI tract using endoscopy. This dataset is made up of 373 videos with 889,372 frames. 343 of those were used for training and 30 (8.7%) were used for testing, which is close to the split used by STTN (Zeng et al., 2020). Note that in testing a limit of 927 frames was set for each video. Even though STTN uses 4603 videos, their frames only reach 681,450 making our pseudo ground truth dataset of a similar and even larger size. In addition, our task of specular highlight inpainting has very narrow video content and similar features as opposed to the various video stream content in the datasets STTN used such as Youtube-VOS (Xu et al., 2018) and DAVIS (Caelles et al., 2018).

To evaluate results and since paired data is available (data with pseudo ground truth), Peak Signal to Noise Ratio (PSNR), Structural Similarity Index (SSIM), and Mean Square Error (MSE) are generated at cropped specularity regions of the pseudo masks $Masks_{Trans}$. Then, the average of the metrics along all the frames in a video is calculated. Since SSIM takes into consideration the relations within windows in a frame using a Gaussian distribution, we first compute the overall frame SSIM and then take the average for the pixels only inside the region of interest. This evaluation process is applied to all testing videos. After that, another averaging step is performed between the values generated from all testing videos in order to get one value for our model.

To assess the generalisability of our system, various datasets were qualitatively evaluated including in-vivo data of gastric endoscopy (Hyper Kvasir and EndoMapper) and ex-vivo porcine data (SERV-CT, SCARED). It is worth mentioning that the model was not fine-tuned on these datasets because they are limited in size.

In addition to that, further analysis is performed on the effect of inpainting endoscopic specular highlights on disparity and optical flow estimation along with feature matching. For the disparity estimation analysis, the ex-vivo porcine SERV-CT dataset is used, which includes accurate ground truth disparities and an evaluation system. The disparity estimation method used is a recent open-source learning-based method called Stereo Transformer proposed by Li et al. (2021).

As for the feature matching analysis, the ORB detector proposed by Rublee et al. (2011) was used to generate keypoints and descriptors. After that, a brute force matcher was utilised. The ratio test as per (Lowe, 1999) was then used to remove low-quality matches. From this, a visual analysis was performed using our dataset, but with the original specularity masks. It is worth mentioning that these computer vision blocks are used in this procedure to illustrate the importance of inpainting in feature matching; however, they can be replaced by any other detector, matcher, and outlier removal methods.

Moving on to the optical flow analysis, the optical flow estimation network Flownet2.0 was used; it was proposed by Ilg et al. (2017) and implemented by Reda et al. (2017). This method can also be replaced by any other optical flow estimation technique to illustrate the importance of inpainting. For the visual assessment, our dataset was also used with the original specularity masks as well as the ex-vivo porcine data with 3D scene ground truth (SCARED dataset).

Along with the visual evaluation, a quantitative assessment was performed to analyse the effect of inpainting specular highlights in endoscopy on sparse feature matching and optical flow. A direct evaluation of optical flow and feature matching was not possible due to the lack of ground truth data. Thus, the matching results, from sparse feature matching or optical flow, were used to generate pose data, which was evaluated with the ground truth pose provided by the SCARED dataset. The SCARED dataset consists of ex-vivo porcine data with 3D scene ground truth. Ex-vivo data was used since available endoscopic data does not have any ground truth information for evaluation; that way, we also assess the extension of our model to other types of surgical data.

To generate and evaluate pose data from the matches, the essential matrix was generated based on the five-point algorithm solver (Nistér, 2004), which relies on the RANSAC algorithm (Fischler and Bolles, 1981). After that, the pose was recovered by decomposing the essential matrix and then verifying possible pose hypotheses using the Chirality check (Nistér, 2004). For this pipeline, an implementation by Gyanesh Malhotra was used (https://github.com/gyanesh-m/Monocular-visual-odometry). After that, relative poses were evaluated with the ground truths and compared between original and inpainted frames. Due to the noise in the ground truth pose information of the SCARED datasets that can appear more dominant with small movements, enough movement between frame pairs had to be ensured. To do that, a moving 20-frame window was used along the sequence to collect frame pairs and evaluate them. This resulted in 715 frame pairs to test on.

It is worth mentioning that the effect of inpainting on feature matching or optical flow and consequently pose estimation might be more visible with endoscopic datasets since the SCARED dataset has very steady and continuous motion and much fewer artefacts that might degrade flow estimation or feature matching. In addition, our model was trained on endoscopy and would naturally perform better on similar testing data. It is also important to note that even though pose estimation is used to assess feature matching and optical flow estimation, it will not be as affected by inpainting as feature matching and optical flow. This is because the RANSAC method used to estimate pose removes outliers, which can make it somewhat robust to some of the artefacts in surgical video streams.

## 5 Results and discussions

In this section, training results are first evaluated and discussed, followed by an ablation study for the importance of the temporal component of our system in the application of specular highlight removal in endoscopy.

The applications of our system are numerous and include the improvement of 3D reconstruction, feature matching, and the estimation of disparity and optical flow. To show our system’s effect on some of these applications, we first started with an analysis for disparity estimation, followed by feature matching, and ended with optical flow.

### 5.1 Model training

In our experiments, several models were generated and compared to reach the best outcome. The models included $Model_{S,R}$, which is trained from scratch (S) on Hyper Kvasir with temporal random masks (R). This training is similar to that of the baseline method, STTN, where diverse videos with temporal random masks are used for training. This model was used to initialise $Model_{T,C}$, which relies on transfer learning (T) and is trained on our pseudo ground truth dataset with pseudo masks $Masks_{Trans}$. In $Model_{T,C}$, (C) refers to correct pseudo masks as opposed to temporal random masks. To show the importance of transfer learning, another model was generated, $Model_{S,C}$, that was trained from scratch on our pseudo ground truth dataset.

**Fig. 5:**
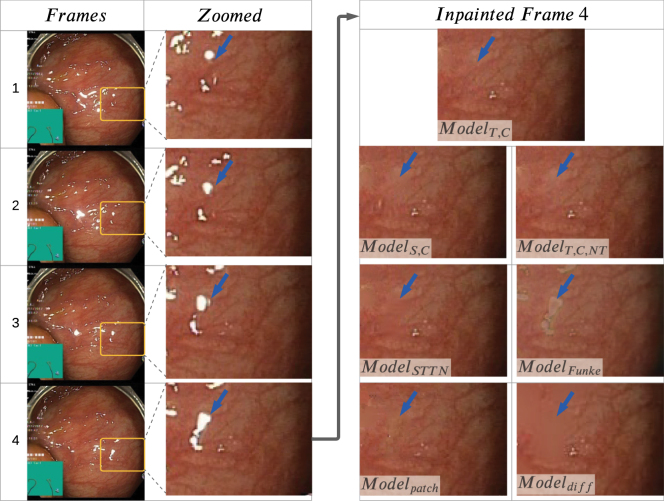
Four consecutive frames are shown on the left. The yellow boxes highlight a region where specularities increase in size. These regions are zoomed in and shown in the second column. Only Frame 4 is inpainted with various methods and results of the zoomed-in region are shown. The blue arrow points towards a prominent occluded vein and how it was inpainted with different methods. It is shown that $Model_{T,C}$ outperformed other models and was able to temporally recover occluded regions that were not occluded in frames 1 and 2.

**Table 1:**
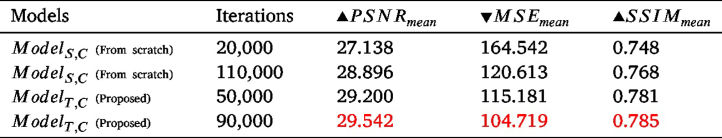
The $PSNR_{mean}$, $MSE_{mean}$, and $SSIM_{mean}$ values for $Model_{S,C}$ and $Model_{T,C}$ at different iterations. $PSNR_{mean}$ and $SSIM_{mean}$ values are better as they increase, which is shown by the ▴ and the opposite can be said about the $MSE_{mean}$ values.

Given that in GANs losses are a trade-off and the optimal loss is reached somewhere between 0 and 1, it cannot be assumed that the model with lower losses is better. Thus, a quantitative and visual analysis is carried out in Fig. 5 and Table 1.

$Model_{T,C}$ was saved at 90,000 iterations, which was decided on the basis of the inflexion point where the loss plots plateau as well as on visual and quantitative analysis. A similar logic was carried out for $Model_{S,C}$, which was used at iteration 110,000 and even though that was not the inflexion point, it did give slightly better results than the results at 20,000 iterations visually and quantitatively.

The quantitative analysis for various iterations of the models can be seen in Table 1. From Table 1, it can be seen in red that the best-performing model according to $PSNR_{mean}$, $SSIM_{mean}$, and $MSE_{mean}$ is $Model_{T,C}$ at 90,000 iterations, indicating the advantage of transfer learning in this application. In addition, in Fig. 5, frame 4 is inpainted by both $Model_{T,C}$ and $Model_{S,C}$. From these figures, it can be seen that the $Model_{T,C}$ learns better temporal inpainting; the red veins are much more prominent and fine-grained for $Model_{T,C}$, which means they have been learnt from previous frames 1 and 2. These images also suggest that even if the quantitative analysis does not show a huge increase in $PSNR_{mean}$, $SSIM_{mean}$, and $MSE_{mean}$ values, it can make a difference in terms of accurate vein depiction. This is due to the fact that the textures in endoscopic frames are very close to each other making any small change count in showing the details better.

Having chosen $Model_{T,C}$ for this system, a comparison between this model and other methods is important. At the time of creating this system, the only available learning-based methods for endoscopic specularities were (Rodríguez-Sánchez et al., 2017) (open source) and Funke et al. (2018) (closed source).

In Funke et al. (2018), specular highlights are inpainted using an end-to-end cycle GAN, however, since the model does not take masks as input, but directly repairs the whole image so as to not have any specularities, we were unable to evaluate it with our pseudo ground truth. That is because the overlaid pseudo masks on the image were not being detected by the network as specularities and thus left without inpainting. That is why, apart from visual comparisons, we have limited the quantitative analysis of this approach (denoted as $Model_{Funke}$) to the downstream computer vision tasks in the upcoming sections.

As for (Rodríguez-Sánchez et al., 2017), specular highlight regions are segmented and then the segmented pixels are fed alone to be inpainted in another network. In other words, their inpainting network only uses limited spatial information only present within specular regions. Thus, this method as well cannot be evaluated using our pseudo ground truth, since no texture information will be present within the overlaid mask to use for inpainting. That is why, we will limit our comparison to only (Funke et al., 2018), which is more relevant to our work since it uses more spatial information.

That is why, using our pseudo ground truth, a comparison is made between our method and non-learning-based approaches. These include a diffusion-based method proposed by Arnold et al. (2010) and a temporal patch-based method proposed by Newson et al. (2014). The models corresponding to these methods will be denoted by $Model_{diff}$ and $Model_{patch}$, respectively.

The visual results of $Model_{diff}$ compared to ours can be seen in Fig. 5, where the inpainted regions are blurry. Thus, it can be seen that no veins were generated with mostly one colour dominating the region. This can also be seen in the quantitative results in Table 2, where our system gives much better results. These results are reasonable since the diffusion method smooths pixel values into the empty regions instead of accurately depicting the missing features as can be done using other methods.

As for $Model_{patch}$, it performed better than $Model_{diff}$, visually and quantitatively, but still not as good as the compared learning-based approaches (Fig. 5, Table 2).

$Model_{Funke}$ was not compared quantitatively, but as can be seen in a visual example it does not inpaint as successfully as our method (Fig. 5). It is worth mentioning that results were obtained with the model that was trained on laparoscopic images and ideally, the model should be retrained with appropriate data and adjusted to fit our data domain. However, this was not done, since the data training procedure is very different than ours requiring creating a new dataset. Their training data is made up of cropped patches with and without specularities. Nonetheless, we do compare further on downstream tasks by using ex-vivo porcine laparoscopic data.

In summary, $Model_{T,C}$ gave the best performance among the various methods according to our quantitative and visual evaluations; it was followed by $Model_{patch}$ then $Model_{diff}$.

**Table 2:**
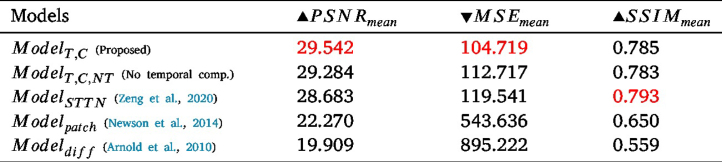
The $PSNR_{mean}$, $MSE_{mean}$, and $SSIM_{mean}$ values for the various trained models. $PSNR_{mean}$ and $SSIM_{mean}$ values are better as they increase, which is shown by the ▴ and the opposite can be said about the $MSE_{mean}$ values.

### 5.2 Ablation study

An ablation study is performed to observe the effect of the temporal component on the output. We compare our proposed model in its original form against the same model but only considering a single frame input (i.e. it does not consider a temporal component). This experiment is denoted as $Model_{T,C,NT}$ (T-Transfer learning, C-Correct Pseudo Masks, NT-No temporal). The visual and quantitative results can be seen in Fig. 5 and Table 2, respectively. It is shown that excluding the temporal component not only gives worse quantitative results but also misses details that can be obtained from previous frames. To elaborate, if one follows the yellow boxes throughout the original frames in Fig. 5, it is clear that the region gets more occluded with every frame, meaning that a temporal component should be able to take advantage of unoccluded regions from previous frames and exploit it during inpainting. This advantage can be seen when one also compares the output for $Model_{T,C,NT}$ and $Model_{T,C}$, where a prominent vein is blurred in $Model_{T,C,NT}$ while its details are visible in $Model_{T,C}$.

The original STTN model (Zeng et al., 2020), trained on Youtube-VOS, $Model_{STTN}$, was also tested on our pseudo ground truth dataset to see the effect of our training methodology. The visual and quantitative results of these models can be seen in Fig. 5 and Table 2, respectively. The results show a visual improvement of our method in Fig. 5 and in Table 2 with PSNR and MSE. However, the SSIM value for this method shows a better score than our temporal and non-temporal methods, which is not aligned with PSNR, MSE, and visual evaluations. This difference in values could be because specular regions can be quite small and SSIM results on a very small amount of pixels should be interpreted with extreme caution and thus might not be appropriate to our case (Nilsson and Akenine-Möller, 2020).

In summary, $Model_{T,C}$ gave the best performance among the various methods according to our quantitative and visual evaluations; it was followed by $Model_{T,C,NT}$, $Model_{STTN}$, $Model_{patch}$, then $Model_{diff}$.

### 5.3 Generalisability

We performed an analysis of the generalisability of our system, without any additional fine-tuning. Our system trained on Hyper Kvasir data was tested on 3 additional datasets with different characteristics. The results of sampled frames can be visualised in Fig. 1 (additional results are provided in the supplementary material). EndoMapper dataset was also used, which is similar to Hyper Kvasir, however, it was acquired on a different site and with different equipment. This data was collected in Clinico Lozano Blesa Hospital using Olympus Europe endoscopic system, whereas Hyper Kvasir was performed in Bærum Hospital using standard equipment from Olympus Europe and Pentax Medical Europe. In general, the results of this data were satisfactory, where veins were uncovered under specularities as seen in yellow in Fig. 1. However, sometimes smaller specularities are inpainted without much detail as seen in red in Fig. 1. This means that the model was able to generalise to other hospitals and equipment, however, the results are not as good as the dataset used in training.

The other two datasets (SERV-CT, SCARED) are of ex-vivo porcine data acquired with a laparoscope. SERV-CT only has keyframes, which makes the temporal component of our system not utilised fully. However, it still gave good results as can be seen in yellow in Fig. 1. The red circles show an error that occurs due to the detection system’s inability to detect saturation. As for the SCARED dataset, the frames are closer, but since it is also ex-vivo data, the colours and textures are different than the trained dataset, which makes the results not detailed as seen in red. However, we can still see some successful inpaintings in the yellow circles. To conclude, our model can be generalised to ex-vivo data with lower-quality results, especially in regions where colours are different than those in in-vivo endoscopy.

### 5.4 Stereo disparity

**Fig. 6:**
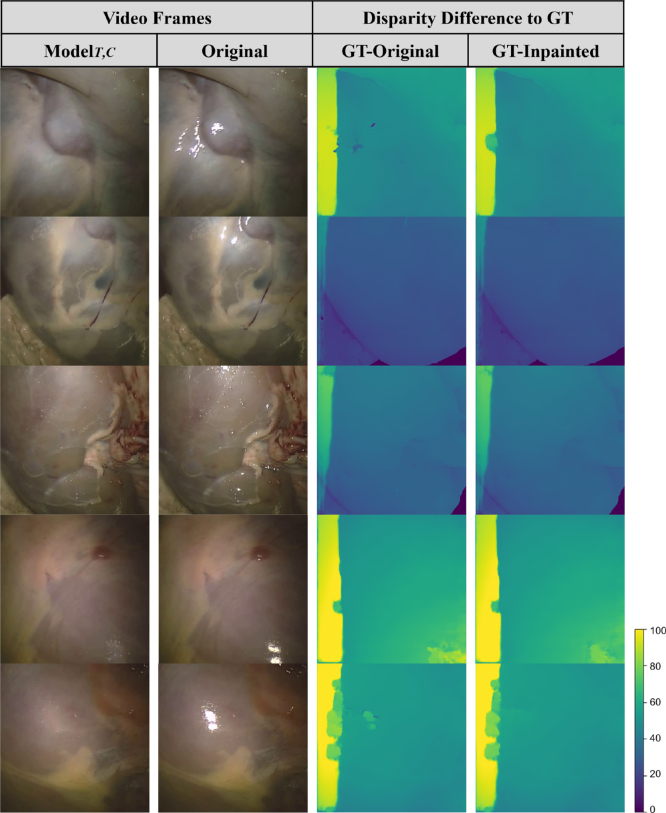
The difference map between ground truth and estimated disparity based on original as well as inpainted frames of $Model_{T,C}$. The last two rows are the highest-scoring frames according to our quantitative results.

In this section, we evaluate the effect of inpainting endoscopic specular highlights on stereo disparity calculation. The SERV-CT evaluation system and dataset, which includes ground truth disparities, are used. As shown in Fig. 6, the disparity generated is closer to the ground truth after inpainting the SERV-CT data using $Model_{T,C}$. Some spurious artefacts in the disparity maps are visibly removed if inpainting is applied. Additionally, the overall disparity values are closer to the ground truth with inpainting.

These disparity results were confirmed by the quantitative evaluation in Table 3 for $Model_{T,C}$. We measure the root mean square error (RMS), the endpoint error (EPE), and the percentage of the disparity image with more than a three-pixel disparity error (bad3% error). The errors are relative to ground truth disparities obtained from CT scanning and Creaform RGB scanning, as indicated in the table. These metrics were only evaluated on the region of the disparity map containing specularities. The evaluations were also done for stereo occlusions SO, where stereo-occluded and non-occluded pixels were evaluated separately. These stereo occlusions are not to be confused with specularity occlusions. Stereo occlusions refer to occlusions occurring due to different viewpoints of the left and right stereo images. The difference between the RMS and EPE of the original versus the inpainted-based disparity estimations were all positive meaning that there was an improvement in the disparity estimation due to inpainting using $Model_{T,C}$.

In particular, some of the highest scoring results, according to our quantitative analysis, can be seen in the last two rows of Fig. 6, where the inpainted results using $Model_{T,C}$ are closer to the original and do not have artefacts due to specularities. Quantitatively, the RMS and EPE are improved by 9.36% and 9.36%, respectively, for the first row and 11.17% and 10.85% for the second row.

**Table 3:**
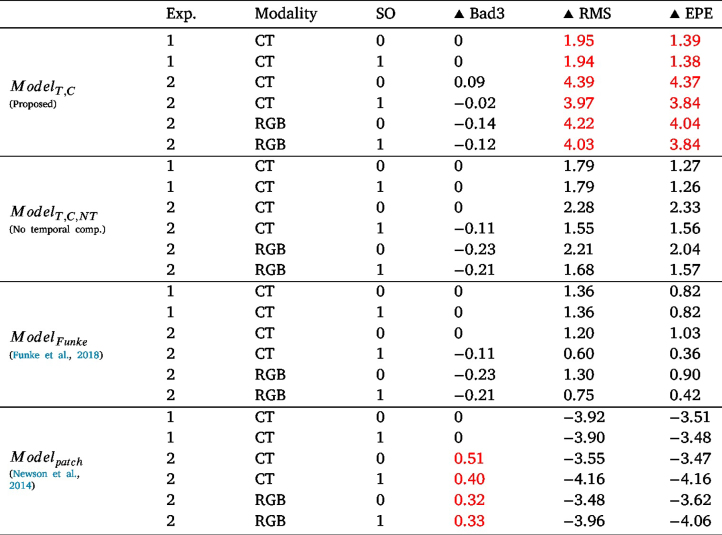
The difference (in %) between quantitative evaluations of disparity generation from original frames, $Disp_{orig}$, and that from inpainted frames using $Model_{T,C}$, $Disp_{inp}$. Two experiments (Exp.) are used with different ground truth modes (Modality) and with and without stereo-occluded pixels (SO).

Even though SERV-CT frames are keyframes, making the temporal component of the model not used to its full potential, the disparity from inpainted frames shows improvement over that from original frames visually and quantitatively.

This disparity evaluation was also performed on other methods and according to the quantitative results (RMS, EPE) in Table 3, the order from best performing method to least is $Model_{T,C}$, $Model_{T,C,NT}$, $Model_{Funke}$, then $Model_{patch}$.

For all disparity estimations, including all inpainting methods and the original without inpainting, the Bad3% error ranges between 99.46% and 100%, which indicates that almost all disparity values estimated in specularity regions even after inpainting are more than 3 pixels apart from the ground truth disparity. The numbers shown in Table 3 are the per cent difference between Bad3% of each inpainting method and the original disparity estimation without inpainting. Since these Bad3% values range between 99.46% and 100%, the per cent difference in the table ranges between 0.51%–0.23%, which is not indicative of any performance conclusion, other than that even after inpainting almost all pixels are still more than 3 pixels apart from the GT disparity.

### 5.5 Sparse feature matching

**Fig. 7:**
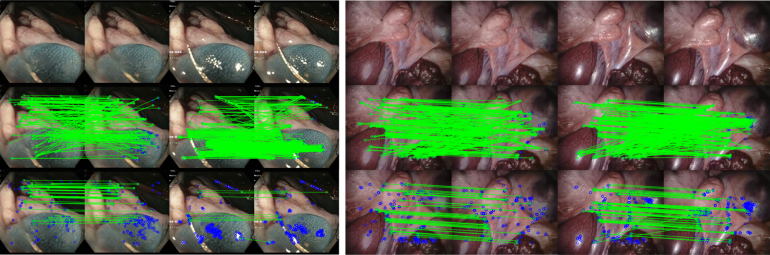
Feature matching comparison between original and inpainted frames using $Model_{T,C}$. Row 1 shows the frames, Row 2 shows the feature matching, and Row 3 shows the matching after filtering outliers. Matches between features are shown in green and the removed low-quality features are shown in blue.

To assess the effect of our method $Model_{T,C}$ on sparse feature matching, we detect and match ORB features (Rublee et al., 2011) on our test dataset. Qualitative results can be visualised in Fig. 7 on the left. By averaging out the number of matches with and without inpainting we can see that inpainting reduces the number of detected features in image pairs by 28.4% on average, but these could be features located on specular highlights that have high gradients but are generally outliers. After outlier filtering using a ratio test, the number of detected matches remains lower for inpainted pairs by 30.8% on average. Since this analysis does not give us any information about the type of features being removed, no conclusions can be made.

To assess the accuracy of these matches, we use them for estimating relative poses between pairs of camera views. Ground truth of camera relative poses is available via the captured robot kinematics as part of the SCARED dataset. The relative pose is estimated with the 5-point algorithm solver within the RANSAC robust estimator (Nistér, 2004). The relative translation and rotation errors (RTE and RRE in degrees) as well as the number of inliers detected by RANSAC were calculated with respect to the ground truth.

**Table 4:**
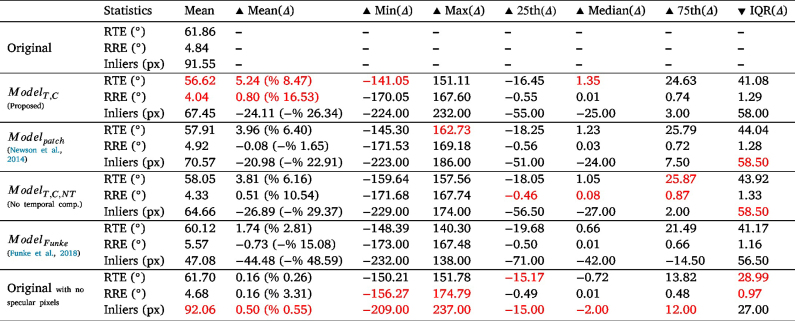
Feature-based pose estimation metrics: Minimum, Maximum, Mean, 25th percentile, Median, 75th percentile, and Interquartile Range (IQR). Metrics with the Δ symbol denote the variation with respect to the baseline.

**Fig. 8:**
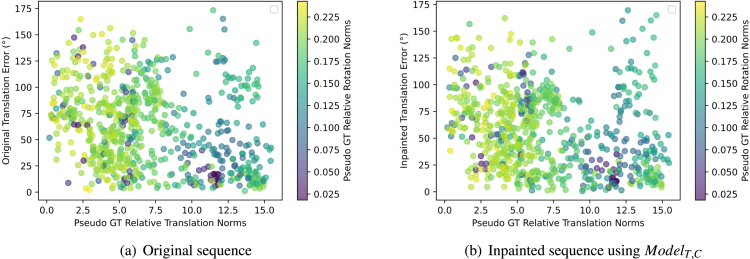
Feature matching analysis: Effect of the ground truth translation and rotation norms on RTE. Low translation norms result in high errors or noise.

**Fig. 9:**
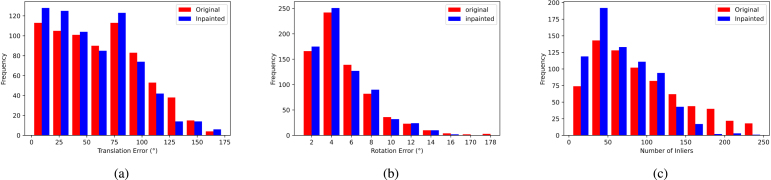
Feature matching analysis: Histograms of RTE, RRE, and RANSAC Inliers for both original and inpainted sequences using $Model_{T,C}$.

A statistical summary of the RTE, RRE, and number of inliers for $Model_{T,C}$ with respect to the baseline is shown in Table 4. For RTE, the interquartile range ([−16.45,24.63]°) is larger on the positive region and the median (1.35°) and mean (% 8.47) fall completely in the positive region. The positive numbers mean that the RTEs for the original sequence are higher than those for the inpainted sequence, which shows that inpainting can help in increasing the accuracy of relative translation estimation, which can be useful for reconstruction and visual SLAM methods.

One can also notice that the mean RTE values are very high in general. After investigating and plotting the effect of the relative translation and rotation norms on the RTE as shown in Fig. 8-(a)(b), a clear effect can be seen between the relative translation and rotation norms and the RTE. This is a well-known problem in relative pose estimation with monocular cameras, where errors of small translations and high rotations are difficult to assess and may be under the precision of robot kinematics used to generate ground truth.

To further visualise these numbers, looking at Fig. 9-(a), we can see that the inpainted sequence has more lower errors (0–50) than the original and less higher errors (100–175).

When comparing RTE values with other methods (Table 4) we can see that according to the mean values the order of best-performing method to least is $Model_{T,C}$, $Model_{patch}$, $Model_{T,C,NT}$, then $Model_{Funke}$. When removing the specular pixels completely from consideration before performing pose estimation, we get the lowest results, showing that for translation estimation using optical flow, inpainting improves the outcome, with the best inpainting method being that proposed in this paper, $Model_{T,C}$.

A similar analysis can be done for the relative rotation error (RRE) of $Model_{T,C}$. However, the RREs are reasonably small with a few outliers. We can see the skewness of the interquartile range ([−0.55,0.74]°), median (0.01°), and mean (% 16.53) to the positive region in Table 4, which indicates an improvement in relative rotation estimation from feature matching after inpainting using $Model_{T,C}$.

This improvement is also shown in Fig. 9-(b), where the inpainted sequence has lower errors (0−5°) than the original. Note that for clarity in Fig. 9-(b), all bars with heights less or equal to 1 are removed.

When comparing RRE values with other methods (Table 4) we can see that according to the mean values the order of best-performing method to least is $Model_{T,C}$, $Model_{T,C,NT}$, $Model_{patch}$ then $Model_{Funke}$ with the last two methods having negative results. Unlike translation, it seems that for rotation, when removing the specular pixels completely from consideration before performing pose estimation, the results are better than those of $Model_{patch}$ and $Model_{Funke}$; This shows that for rotation not all inpainting methods improved the outcome. The best inpainting method was that proposed in this paper, $Model_{T,C}$.

Finally, the numbers in Table 4 show that the original sequence has a higher number of inliers than the inpainted one (with $Model_{T,C}$) with an interquartile range ([−55.00,3.00]pixels), which is much larger on the negative region, and a median (−25.0 pixels) and mean (–% 26.34) falling completely in the negative region. This means that the original sequence has more inliers. This can indicate that fewer pixels are wrongly identified as inliers.

This is also shown in Fig. 9-(c), where the inpainted sequence has more lower number of inliers (0–125) and less of the higher number of inliers (125–250) than the original.

When comparing inliers with other methods (Table 4) we can see that according to the mean values, all inpainting methods have a lower number of inliers than the original sequence. When removing the specular pixels completely from consideration before performing pose estimation, we get the highest number of inliers (highest Mean(Δ)). However, this method did not perform as high on the RRE and RTE metrics, this can be due to an increase in outliers in the face of inliers or that the inliers filtered by RANSAC might have been false positives.

To conclude, feature matching was improved the most using our inpainting method $Model_{T,C}$. This was evaluated visually as well as quantitatively through pose estimation and the assessment of RTE, RRE, and RANSAC inliers. According to this assessment, the highest scoring results that improved pose estimation by 92.4% can be shown in Fig. 7 on the right. It can be seen that the original frame pair matches are much more reliant on specular highlights than the inpainted frame pair matches.

### 5.6 Optical flow

**Fig. 10:**
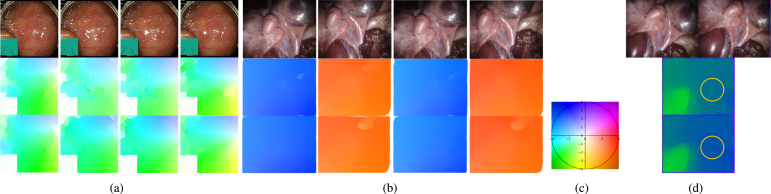
(a) From four consecutive frames of the Hyper Kvasir dataset (First Row), optical flow is estimated directly (Second Row) and after inpainting the frames using $Model_{T,C}$ (Third Row). Inpainting improved the flow estimation results by making it smoother and more homogeneous. (b) From four consecutive frames of the SCARED dataset (First Row), optical flow is estimated directly (Second Row) and after inpainting the frames using $Model_{T,C}$ (Third Row). It is not clear if inpainting improved the flow estimation results. The SCARED dataset used differs from the training dataset (Hyper Kvasir) in its surgical procedure performed. (c) Optical flow colour wheel. (d) The estimated optical flow gave the highest improvement with inpainting (Third Row) according to our quantitative assessment. A removed artefact can be seen in the yellow circle. The colour map was changed in this example for visual clarity.

The effect of our method $Model_{T,C}$ on optical flow, using FLOWNET2.0, can be qualitatively visualised in Fig. 10 for Hyper Kvasir and SCARED datasets. It can be seen that the optical flow generated from the inpainted frames is smoother, and has fewer spurious artefacts. More results are provided in the supplementary material.

Similar to the previous section, we evaluate the optical flow results quantitatively via relative pose estimation between pairs of frames, using the same algorithmic and validation pipeline.

The RTE, RRE, and number of inliers were statistically summarised in Table 5 for $Model_{T,C}$ with respect to the baseline. The RTE interquartile range ([−7.20,10.80]°) is skewed to the positive region. In addition, the median (1.65°) and mean % 3.64 are positive, meaning that inpainting using $Model_{T,C}$ increased translation estimation accuracy by % 3.64 on average.

**Fig. 11:**
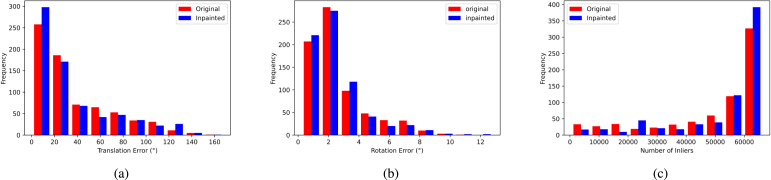
Optical flow analysis: Histograms of RTE, RRE, and RANSAC Inliers for both original and inpainted sequences using $Model_{T,C}$.

To further visualise these numbers, looking at Fig. 11-(a), we can see that the inpainted sequence has more lower errors (0–20) than the original and no apparent increase or decrease in the higher errors (20–175).

**Table 5:**
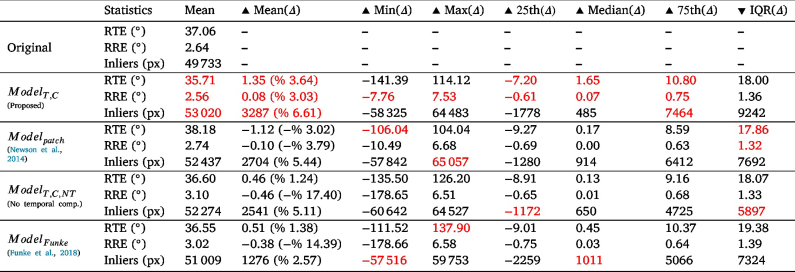
Optical-flow-based pose estimation metrics: Minimum, Maximum, Mean, 25th percentile, Median, 75th percentile, and Interquartile Range (IQR). Metrics with the Δ symbol denote the variation with respect to the baseline.

**Fig. 12:**
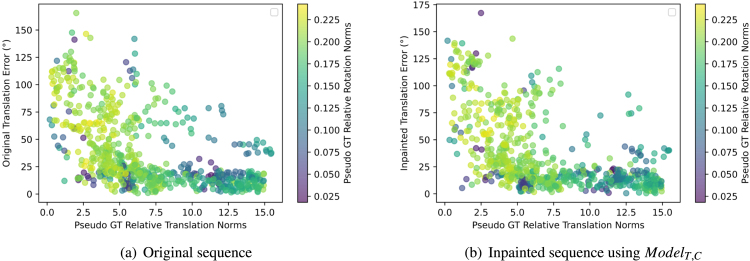
Optical flow analysis: Effect of the ground truth translation and rotation norms on RTE. Low translation norms result in high errors or noise.

From Table 5, the RTE and RRE improvements due to inpainting are not as high as those with feature matching. Similar to feature matching, the relationship between translation errors and translation and rotation norms shows a high correlation in Fig. 12.

When comparing RTE values with other methods (Table 5) we can see that according to the mean values the order of best performing method to least is $Model_{T,C}$, $Model_{Funke}$, $Model_{T,C,NT}$, then $Model_{patch}$. The mean RTE value is negative for $Model_{patch}$, which means that for translation estimation using optical flow, learning-based inpainting improves the outcome, whereas temporal patch-based methods do not. The best inpainting method is that proposed in this paper, $Model_{T,C}$.

For relative rotation errors, we look at the Δ RRE statistics in Table 5 for $Model_{T,C}$, where the interquartile range ([−0.61,0.75]°), mean (%3.03), and median (0.07°) with respect to the baseline are all skewed to the positive region. This can also be seen in Fig. 11-(b).

When comparing RRE values with other methods (Table 5) we can see that according to the mean values the order of best-performing method to least is $Model_{T,C}$, $Model_{patch}$, $Model_{Funke}$, then $Model_{T,C,NT}$. Only the proposed method improved results, whereas all the others had higher RRE errors when compared to using optical flow without inpainting.

Finally, the numbers in Table 5 for $Model_{T,C}$ show that the original sequence has a lower number of inliers than the inpainted one with an interquartile range ([−1778,7464]pixels), which is larger on the positive region, and a median (485 pixels) and mean (% 6.61) falling completely in the positive region. This can also be seen in Fig. 11-(c). According to the mean values, the order of best performing method to least is $Model_{T,C}$, $Model_{patch}$, $Model_{T,C,NT}$, then $Model_{Funke}$. This means that $Model_{T,C}$ was able to generate the highest number of inliers. This is notoriously different from sparse feature matching. This can be due to the dense nature of optical flow, where more pixels, such as the inpainted pixels, are included as inliers. This does not happen with sparse feature matching, since only sparse important features are detected and inpainting makes the specular regions less significant.

To conclude, the overall motion estimation accuracy with optical flow was improved the most using our inpainting method $Model_{T,C}$. However, the improvement with optical flow is not as high as that with sparse feature matching. This can be due to optical flow providing dense correspondences and offering a surplus of information when compared to sparse features; this surplus makes RANSAC more effective at removing outliers caused by spurious artefacts. We also note that our method has not been fine-tuned on laparoscopic data (SCARED dataset), and therefore further improvements could be obtained with additional surgery-specific training data. This is consistent with the more visible qualitative improvements in the Hyper Kvasir data, which is in the same domain as our training data.

According to the quantitative analysis, the highest scoring results that improved pose estimation by 93.8% using $Model_{T,C}$ can be shown in Fig. 10-(d). Even though quantitatively these are the highest scoring results, since the frames used are 20 frames apart, visually it is not very clear, however, a removed artefact can be seen in the yellow circle.

## 6 Limitations

The main limitation of the proposed system is its reliance on the detection method. This can sometimes miss some specularities and be thrown off by text and diagram information overlaid by endoscopic camera interfaces. This can be seen for example in Fig. 3-(a)(c), where the text and green square are detected as specularities and are later on inpainted. This can affect the learning process of the model and limit its ability to find similarities between occluded regions of different frames. In the future, the system can be expanded to include a detection system of static screen objects and remove them from consideration.

## 7 Conclusion

In this paper, a system was proposed to inpaint specular highlights in MISD videos. An endoscopic pseudo ground truth dataset was generated after which a model was trained using temporal GANs and transfer learning to produce inpainted frames based on spatial–temporal information. The qualitative and quantitative results of the system showed improvement over other methods. An ablation study was also carried out to show the importance of the temporal component and transfer learning in generating enhanced frames. In addition, the effect of this system on various applications including feature matching, disparity estimation, and optical flow prediction was shown to be significant. This effect was also used to show that the performance of our method supersedes other methods.

## Declaration of competing interest

The authors declare the following financial interests/personal relationships which may be considered as potential competing interests: Rema Daher reports financial support was provided by Engineering and Physical Sciences Research Council. Francisco Vasconcelos, Danail Stoyanov reports financial support was provided by Engineering and Physical Sciences Research Council. Francisco Vasconcelos, Danail Stoyanov reports financial support was provided by Wellcome Trust. Rema Daher reports financial support was provided by Wellcome Trust. Rema Daher reports financial support was provided by Horizon Europe. Rema Daher reports financial support was provided by Amazon Web Services, Inc. Danail Stoyanov reports a relationship with Odin Vision Ltd that includes: equity or stocks. Danail Stoyanov reports a relationship with Panda Surgical Ltd that includes: equity or stocks. Danail Stoyanov reports a relationship with Medtronic plc that includes: employment.

## Data Availability

https://github.com/endomapper/Endo-STTN

## Appendix A Supplementary data

Video S1 Video results for specular highlight inpainting and optical flow estimation directly and after inpainting.

MMC S2 Visual results for optical flow estimation directly and after inpainting.
