# Supplementary material for: A Temporal Learning Approach to Inpainting Endoscopic Specularities and Its Effect on Image Correspondence
Source: Med Image Anal. 2023 Dec;90:102994. doi: 10.1016/j.media.2023.102994 (PMC10958122; doi:10.1016/j.media.2023.102994)

Supplementary Material

In the following document, the supplementary material is described and displayed.

The specular highlight inpainting system is trained on a portion of the Hyper-Kvasir dataset (upper and lower gastric endoscopy) and tested on unseen Hyper-Kvasir images and other datasets without any additional fine-tuning, including in-vivo colonoscopy (private data) and ex-vivo porcine laparoscopy (SERV-CT, SCARED). The video results can be seen in the attached video “SupplementaryVideo.mp4”, where the output is only shown on one sequence from each dataset and the optical flow video results with and without inpainting are shown. The video output is of one sequence from the Hyper-Kvasir testing data.

To generate the optical flow from the SCARED dataset, a moving 20 frames window was used. This results in 715 disconnected optical flow images. Since the images are not continuous, a video can not be generated from them. That is why, in this document multiple frames are shown with their corresponding optical flows with and without inpainting.

Section **A.1** shows the optical flow results that were slightly degraded with inpainting. Section **A.2** shows the most prevalent output that shows no apparent change with optical flow when the frames where inpainted. On the contrary, only few frames showed obvious change when inpainted; those that improved with inpainting can be shown in Section **A.3** and those that were degraded with inpainting can be seen in Section **A.4**.

A.1 – Slight Degradation With Inpainting

Original Frames	Optical Flow	Inpainted Frames	Optical Flow

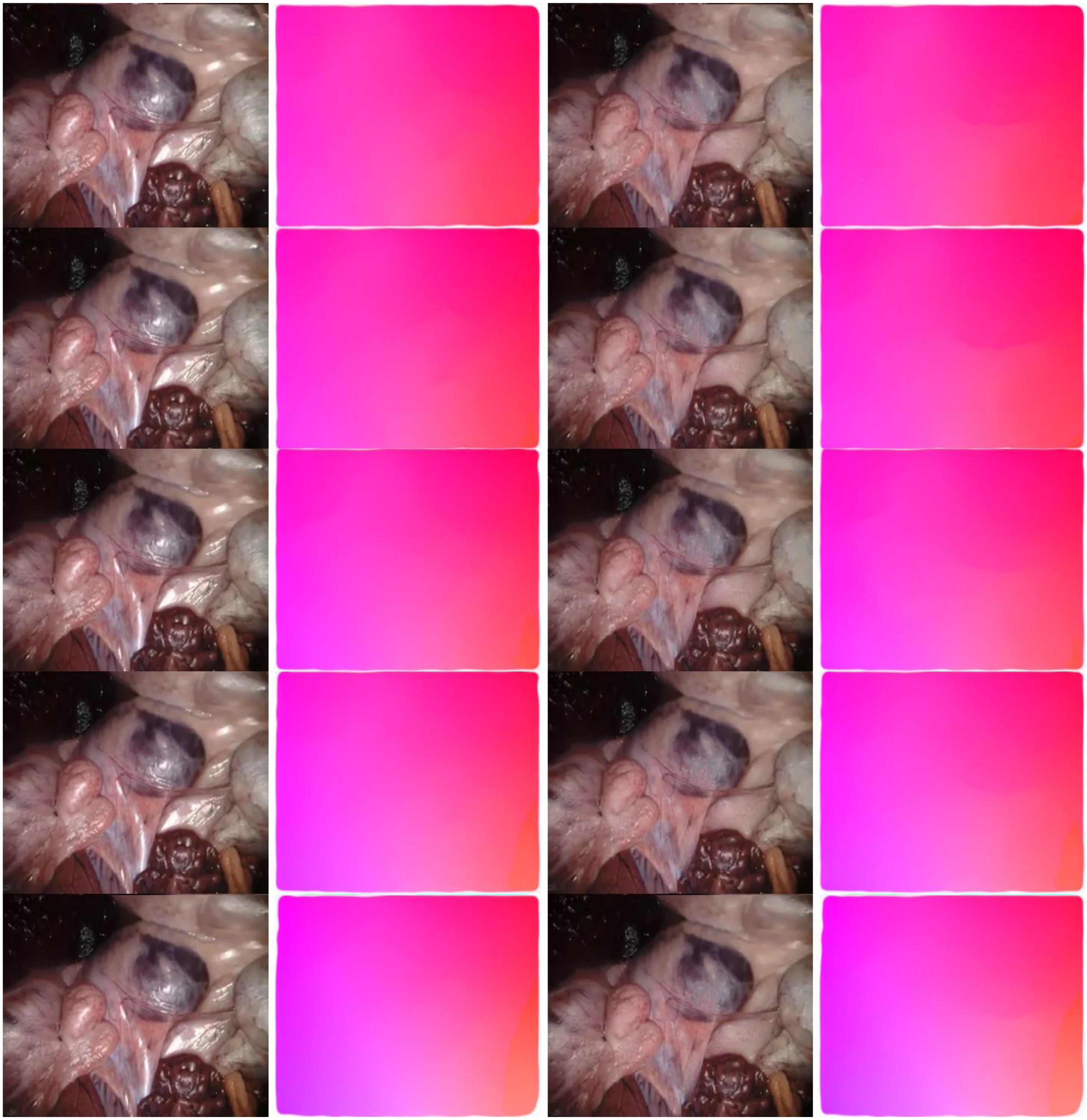

A.2 – No Apparent Change With Inpainting

Original Frames | Optical Flow | Inpainted Frames | Optical Flow

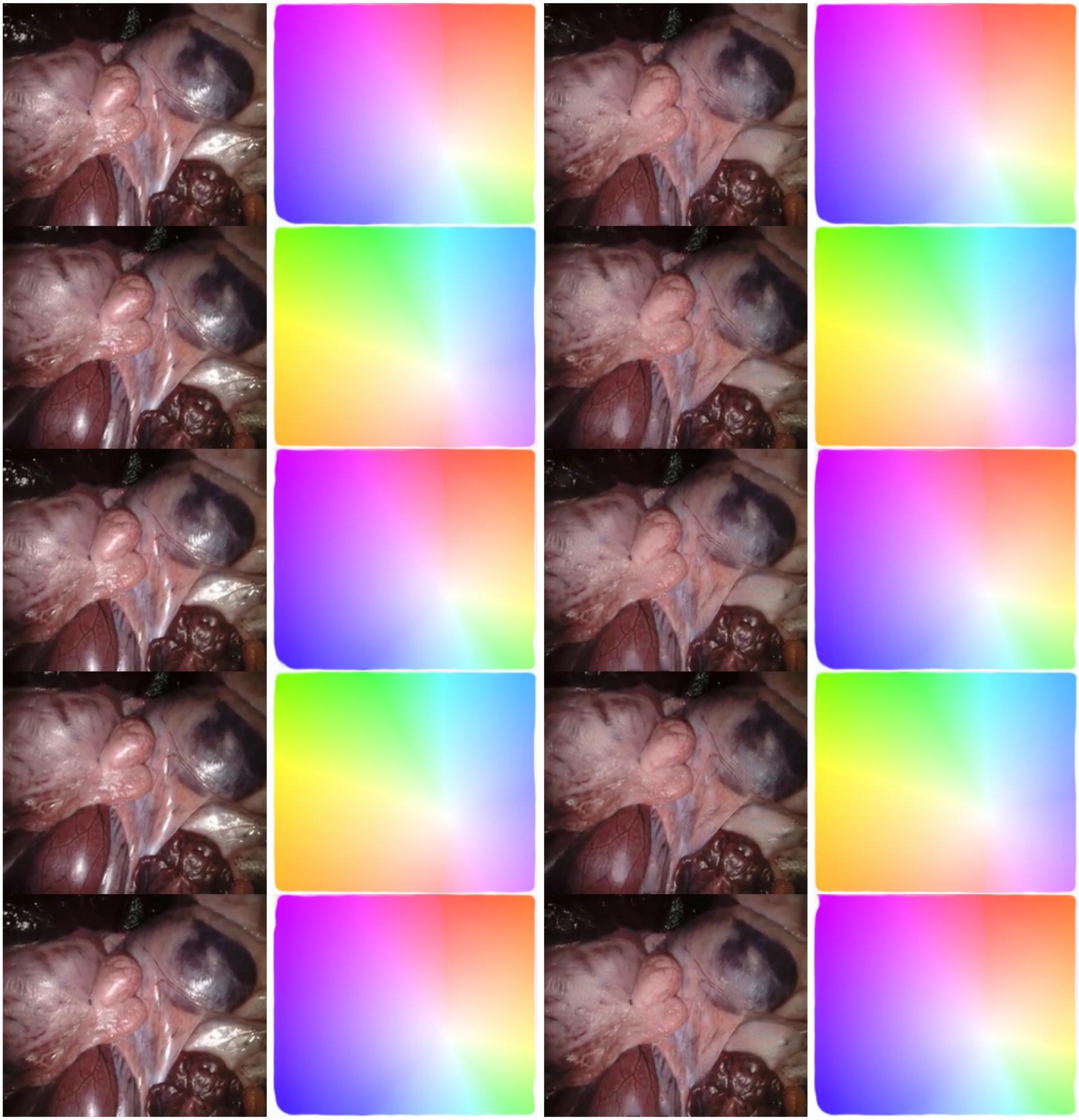

A.3 – Improvement With Inpainting

Original Frames	Optical Flow	Inpainted Frames	Optical Flow

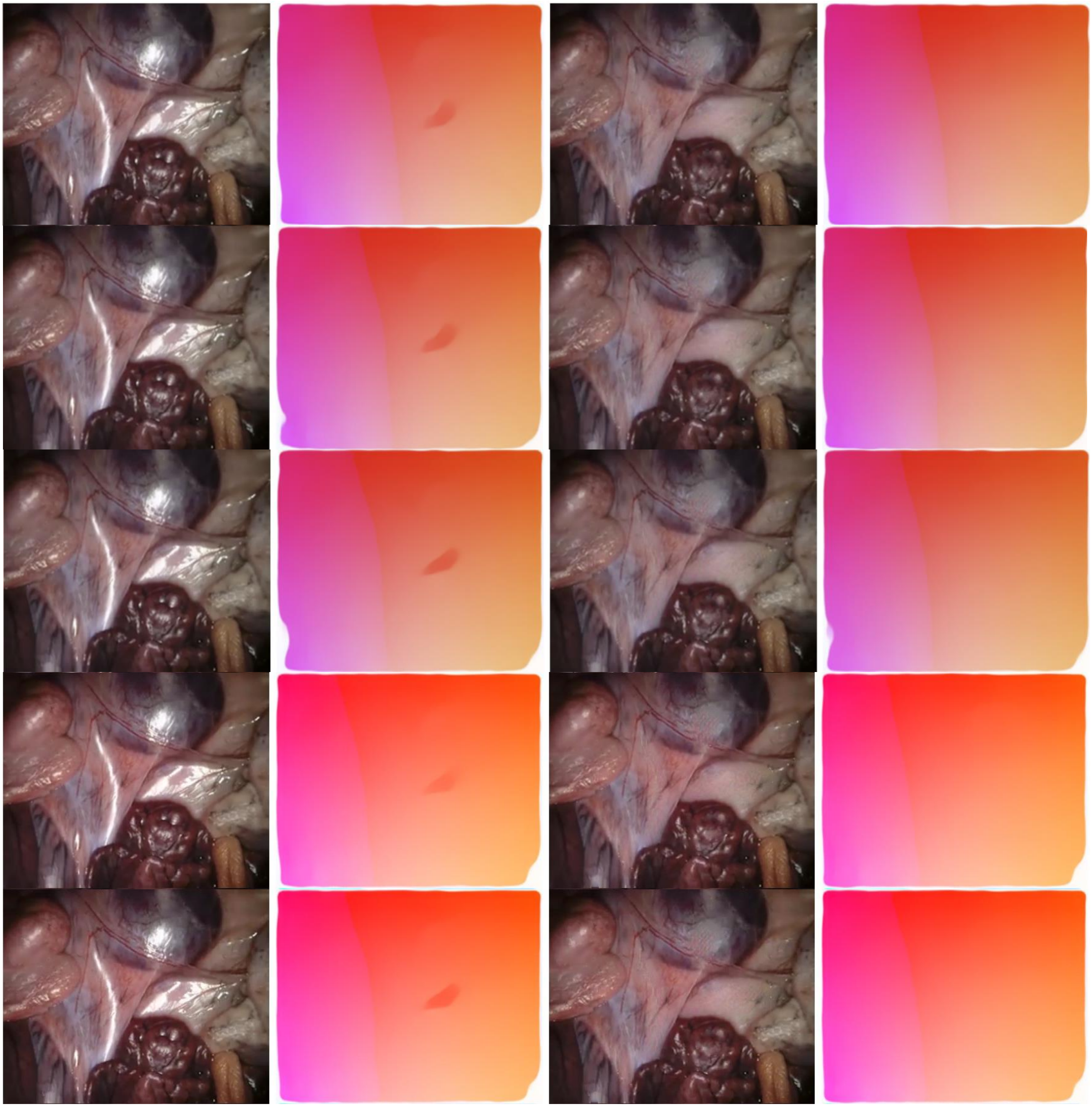

A.4 – Degradation With Inpainting

Original Frames	Optical Flow	Inpainted Frames	Optical Flow

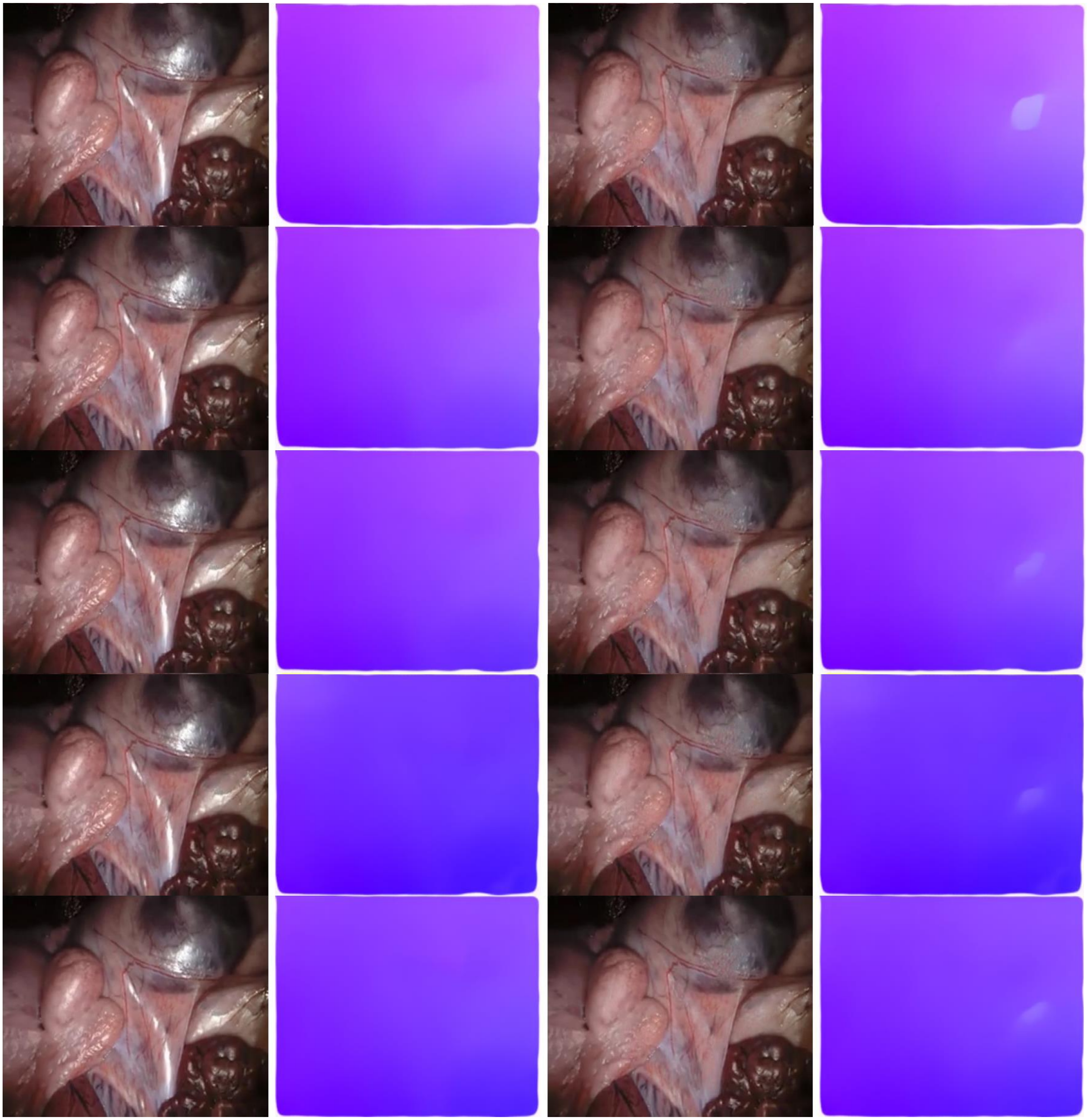

Supplement: MMC S2 — Visual results for optical flow estimation directly and after inpainting. [file mmc2.pdf]
